# Identification and Characterization of Lipid Droplet-Associated Protein (LDAP) Isoforms from Tung Tree (*Vernicia fordii*)

**DOI:** 10.3390/plants14050814

**Published:** 2025-03-05

**Authors:** Alyssa C. Clews, Payton S. Whitehead, Lingling Zhang, Shiyou Lü, Jay M. Shockey, Kent D. Chapman, John M. Dyer, Yang Xu, Robert T. Mullen

**Affiliations:** 1 Department of Molecular and Cellular Biology, University of Guelph, Guelph, ON N1G 2W1, Canada; clewsa@uoguelph.ca; 2 BioDiscovery Institute, Department of Biological Sciences, University of North Texas, Denton, TX 76203, USA; paytonwhitehead@my.unt.edu (P.S.W.); kent.chapman@unt.edu (K.D.C.); 3 Key Laboratory of Plant Germplasm Enhancement and Specialty Agriculture, Wuhan Botanical Garden, Chinese Academy of Sciences, Wuhan 430074, China; zhanglingling@wbgcas.cn; 4 State Key Laboratory of Biocatalysis and Enzyme Engineering, School of Life Sciences, Hubei University, Wuhan 430062, China; 20190013@hubu.edu.cn; 5 United States Department of Agriculture, Agricultural Research Service, Southern Regional Research Center, New Orleans, LA 70124, USA; jay.shockey@usda.gov (J.M.S.); john.dyer@usda.gov (J.M.D.)

**Keywords:** lipid droplets, α-eleostearic acid, lipid-droplet-associated protein (LDAP), *Vernicia fordii*, tung tree, triacylglycerol, unusual fatty acid

## Abstract

Lipid droplets (LDs) are cytoplasmic organelles responsible primarily for the storage of neutral lipids, such as triacyclglycerols (TAGs). Derived from the endoplasmic reticulum bilayer, LDs are composed of a hydrophobic lipid core encased by a phospholipid monolayer and surface-associated proteins. To date, only a relatively few LD ‘coat’ proteins in plants have been identified and characterized, most of which come from studies of the model plant *Arabidopsis thaliana*. To expand our knowledge of the plant LD proteome, the LD-associated protein (LDAP) family from the tung tree (*Vernicia fordii*), whose seeds are rich in a commercially valuable TAG containing the conjugated fatty acid α-eleostearic acid (C18:3Δ^9*cis*,11*trans*,13*trans*^ [α-ESA]), was identified and characterized. Based on the tung tree transcriptome, three *LDAP* isoforms (*VfLDAP1-3*) were elucidated and the encoded proteins distinctly clustered into three clades along with their respective isoforms from other angiosperm species. Ectopic expression of the *VfLDAPs* in *Nicotiana benthamiana* leaves revealed that they localized specifically to LDs and influenced LD numbers and sizes, as well as increasing TAG content and altering TAG fatty acid composition. Interestingly, in a partially reconstructed TAG-ESA biosynthetic pathway, the co-expression of *VfLDAP3* and, to a lesser degree, *VfLDAP2*, significantly increased the content of α-ESA stored within the LDs. These results suggest that the VfLDAPs can influence the steady-state content and composition of TAG in plant cells and that certain LDAP isoforms may have evolved to more efficiently package TAGs into LDs containing unusual fatty acids, such as α-ESA.

## 1. Introduction

The tung tree (*Vernica fordii*) is a medium-sized deciduous tree from the *Euphorbiaceae* family that has significant economic importance due to the industrial usage of its seed oil [1,2]. Tung seed oil is comprised primarily of triacylglycerols (TAGs) that are enriched in α-eleostearic acid (C18:3Δ^9*cis*,11*trans*,13*trans*^ [α-ESA]), which accounts for approximately 80% of its fatty acid composition [3,4,5]. α-ESA is an unusual conjugated fatty acid that readily oxidizes to form a resilient biopolymer. This “drying” quality makes ESA a valuable component in varnishes, paints and other coatings [5,6,7]. α-ESA also has bioactive properties with potential health applications [2,8,9]. Despite the high value and demand for tung oil, its production is limited by the agronomic properties of the tung tree, including limited geographical growing areas and the length of time it takes between planting of seedlings and generation of a productive crop [2,3]. As a result, there is significant interest in understanding the molecular mechanisms involved in tung oil biosynthesis, with the goal of producing α-ESA-enriched TAGs (TAG-ESA) in other higher-yielding oilseed crop platforms [5,10,11].

To date, several of the enzymes involved in TAG-ESA biosynthesis have been identified and characterized from *V. fordii*. Perhaps the most notable among these are FATTY ACID DESATURASE X (VfFADX) and DIACYLGLYCEROL ACYLTRANSFERASE 2 (VfDGAT2) [12,13,14]. VfFADX is an endoplasmic reticulum (ER) membrane-bound, divergent form of the FATTY ACID DESATURASE 2 (FAD2) enzyme that synthesizes ESA from linoleic acid (C18:2Δ^9*cis*,12cis^) while bound to the *sn*-2 position of phosphatidylcholine [15,16]. The FAD2 enzyme is typically involved in the synthesis of linoleic acid, which is common to all plants, but other, divergent FAD2 enzymes are known to be responsible for the synthesis of numerous, structurally diverse or so-called ‘unusual’ fatty acids that accumulate in seed oils of many plants [15]; also see [17] for a recent review of plant FADs in general. VfDGAT2, which also localizes to the ER membrane, synthesizes TAG by transferring ESA from an acyl-CoA donor to the *sn*-3 position of diacylglycerol [12]. Consequently, both enzymes (i.e., VfFADX and VfDGAT2) have been utilized in previous bioengineering strategies aimed at the transgenic production of TAG-ESA [14]. However, the accumulation of ESA in the oil of transgenic seeds and other tissues is far less than in tung oil [14], likely because tung TAG-ESA metabolism requires other enzymes and proteins for not only the proper biosynthesis of ESA and TAG-ESA, but also the proper compartmentalization and storage of TAG-ESA in lipid droplets (LDs).

LDs are found in all organisms and consist of a neutral lipid core, which primarily contains TAGs, surrounded by a phospholipid monolayer and various surface-associated proteins. Based on current working models for eukaryotes [18,19,20], the biogenesis of LDs begins at the ER membrane, where biosynthetic enzymes, such as acyltransferases, produce TAGs that accumulate to form a “lipid lens”, which grows within the ER membrane bilayer. Eventually, a nascent LD buds off the surface of the ER into the cytoplasm or, alternatively, remains physically connected to the ER allowing it to rapidly grow and shrink in response to the needs of the cell/organism. Overall, LD biogenesis is a highly orchestrated, stepwise process that involves numerous proteins, including the ER membrane-bound SEIPIN, which helps determine the site of LD formation, the vectorial budding of LDs towards the cytosol, and facilitates the transfer of neutral lipids into the growing LD. Other proteins, such as the PERILIPINs (PLINs) in animals and OLEOSINs in plants, are recruited to coat nascent LDs to help stabilize the LD monolayer, promote membrane curvature, and/or prevent LD-LD fusion.

In plants, the elucidation of the LD coat proteome is an ongoing field of study. Numerous LD coat proteins haven been discovered in the last decade with a range of functions capable of influencing LD morphology and/or associated-lipid metabolism; for more information, refer to the recent review by Guzha et al [19]. Amongst the predominant LD coat proteins are the OLEOSINs and LIPID DROPLET-ASSOCIATED-PROTEINs (LDAPs), with LDAPs being expressed in seeds as well as most vegetative cell types (i.e., leaves, stems, etc.), and OLEOSINs expressed predominantly in seeds and pollen [19,21,22,23,24]. Over-expression of *OLEOSIN*s or *LDAP*s in transgenic plants often increases LD abundance, which might be due to enhanced LD biogenesis and/or decreased LD turnover [19,21,25,26]. Therefore, both groups of proteins represent attractive targets for bioengineering strategies aimed at increasing oil content in transgenic plants [27,28,29]. However, to date, only the OLEOSINs have been employed in these types of bioengineering study; the ectopic expression of *OLEOSINs* in transgenic seeds or leaves results in an increase in oil content and changes in the ratio of endogenous plant fatty acids [30,31,32,33]. By contrast, for the LDAPs, there is significantly less information on their ability to increase oil content and/or modify fatty acid composition in (transgenic) plants [21,34].

Here, we identified and characterized the LDAP family from the tung tree and explored their potential for modifying TAG content and composition in transient expression assays, including an increase in transgenic TAG-ESA production. Three tung tree LDAP isoforms (VfLDAP1-3) were identified that exhibit an overall similarity in polypeptide sequences and predicted structural elements to each other as well as with LDAPs from other plant species. The VfLDAPs localized specifically to LDs in *Nicotiana benthamiana* leaves and their ectopic expression led to an increase in LD numbers and, albeit to a lesser degree, their sizes. Furthermore, we demonstrate that ectopic expression of individual *VfLDAP*s in *N. benthamiana* leaves differentially altered TAG fatty acid composition, and when co-expressed with *VfFADX* and *VfDGAT2*, which promote TAG-ESA biosynthesis, *VfLDAP2* and *VfLDAP3* supported an increase in the accumulation of TAG-ESA in LDs. Taken together, our results expand the understanding of LDAPs in proper packaging of TAG into LDs and provide potential tools for future usage in bioengineering strategies aimed at production of value-added lipids in plants.

## 2. Results

### 2.1. Tung Has Three Distinct LDAP Isoforms

**Figure 1:**
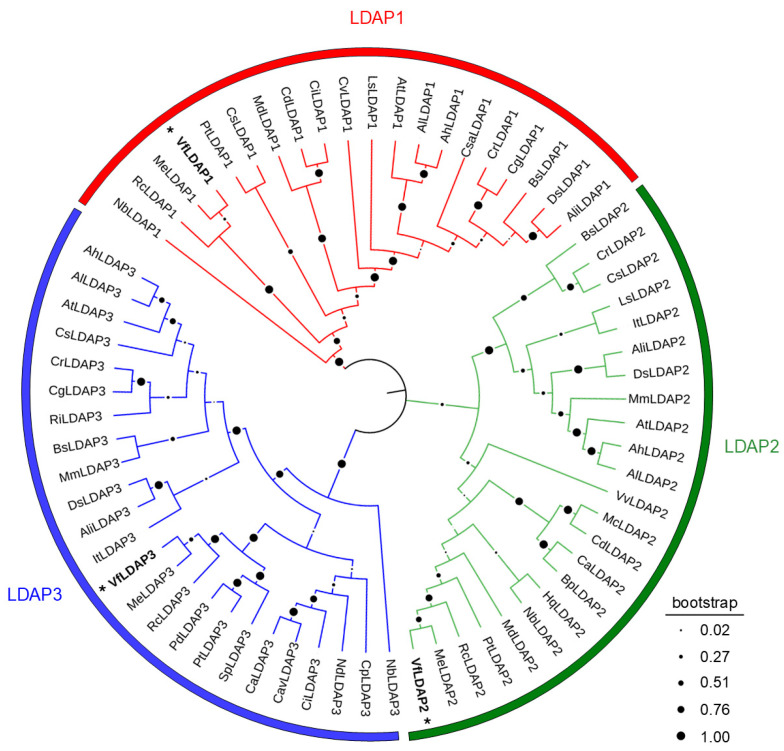
A consensus maximum-likelihood phylogenetic tree (n = 100) depicting the relationship of VfLDAPs and other LDAP homologs from various species in Angiospermae. Sequences used for phylogenetic analysis were the top ten homologs identified in pBLAST searches of the angiosperm species currently available at the Phytozome database, using the tung and Arabidopsis LDAP-deduced amino acid sequences as queries; refer to Table S1 for deduced amino acid sequences and annotations of all the LDAP homologs shown in the phylogenetic tree. Bootstrap values are depicted by circles at the bases of individual notes, with their size proportional to their numerical value (refer to embedded key). Note that the tree consists of three major clades, corresponding to the isoforms for LDAP1 (red), LDAP2 (green) and LDAP3 (blue); the tung LDAPs in each clade are bolded and indicated with asterisks.

**Figure 2:**
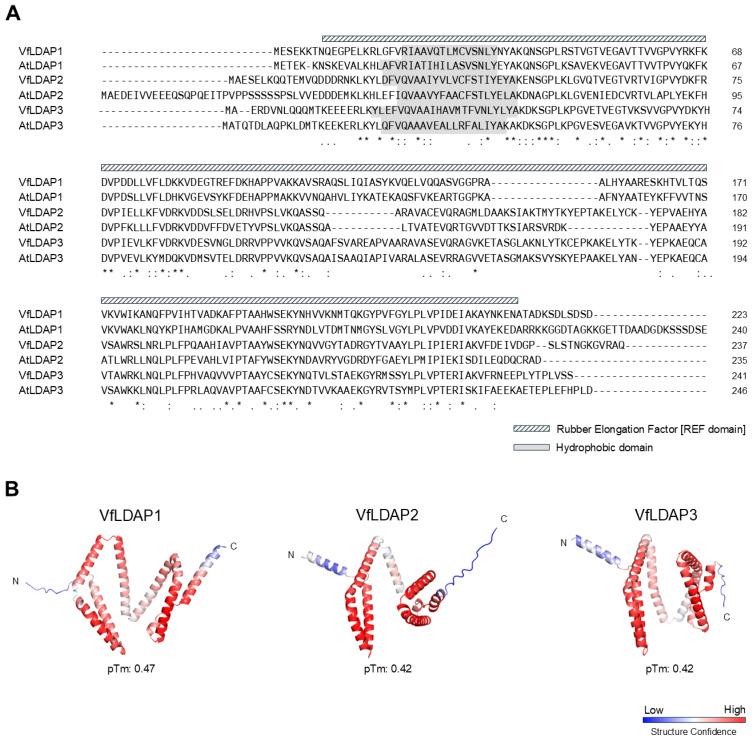
Overview of VfLDAP-deduced amino acid sequences and predicted protein structures. (**A**) Deduced polypeptide sequence alignments of tung and Arabidopsis LDAPs generated with Clustal Omega EMBL-EBI. Identical amino acid residues, conserved substitutions, and semi-conserved substitutions are indicated with asterisks, colons, and periods, respectively. Numbers to the right of each row of sequences represent specific amino acids for each protein. The sequences corresponding to the REF domain in VfLDAP1 (as assessed by the InterPro database [Pfam: PF05755.17]) is indicated with a hatched box above the alignment and corresponds to the approximate position of the REF domains in the other LDAPs. The predicted hydrophobic regions near the N-termini of the LDAPs (based on TMHMM) are shaded in grey, as indicated by the key. (**B**) Three-dimensional structures of the tung LDAPs, as predicted by AlphaFold. The N- and C-termini for each protein are indicated, and the structures are coloured according to prediction confidence; with blue, white, and red colouring representing low, neutral, and high structural confidence, respectively (refer to key). The predicted template modelling (pTM) value for each protein (provided by AlphaFold) is also presented.

To identify putative LDAPs from tung, the cDNAs of the respective Arabidopsis *LDAPs* (*AtLDAP1*, *2* and *3*) [21,35] were used as queries in BLAST (Basic Local Alignment Search Tool) searches of the tung transcriptome database [3]. Overall, three putative tung LDAP isoforms (VfLDAPs) were identified and, based on a maximum-likelihood phylogenetic tree that included LDAP homologs from various other angiosperm species, the VfLDAPs discretely clustered with either LDAP1, 2, or 3 proteins from other species, and, thus, were designated VfLDAP1, 2, and 3 (Figure 1), respectively. As shown also in Figure 1, the VfLDAPs are closest in sequence identity to the LDAPs from cassava (*Manihot esculenta*) and castor bean (*Ricinus communis*), which, like tung, belong to the order Malpighiales. Comparison of the deduced VfLDAP amino acid sequences (along with those of the AtLDAPs) indicated that VfLDAP1 shares 42.0 and 39.7% sequence identity with VfLDAP2 and 3, respectively, whereas VfLDAP2 and 3 are more similar to each other, with a 62.0% amino acid sequence identity (Figure 2A). Consistent with this, the LDAP2 and 3 isoforms in other plant species including Arabidopsis, are also generally more similar in terms of their sequence identity compared to their LDAP1 counterparts (Figure 2A and Figure S1). All three VfLDAPs, along with their homologs in Arabidopsis, also share a number of general physicochemical properties, including their overall length (i.e., ~250 amino acids long), a rubber elongation factor (REF) domain, and a single, putative N-terminal hydrophobic region (Figure 2A). Further, AlphaFold-predicted structural models indicate that all three VfLDAPs, again like their homologs in other plant species, are generally similar, consisting of 8–10 α-helices and 1–3 C-terminal β-sheets (Figure 2B and Figure S2), although they have overall low structure prediction confidence, especially in the N- and C-terminal regions which exhibit the most variability in their predicted structures.

### 2.2. VfLDAPs Are Differentially Expressed in Various Tung Tree Organs and Developmental Stages

**Figure 3:**
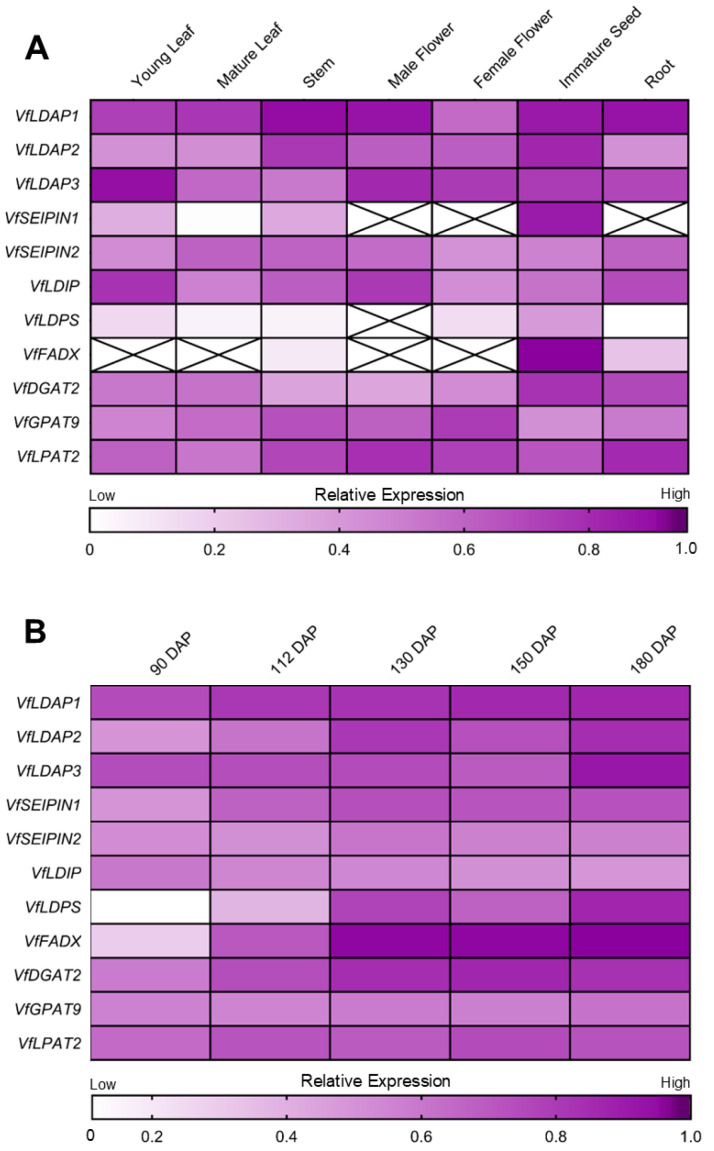
Expression profiles of *VfLDAP* genes in comparison to other selected tung lipid metabolism and LD biogenesis genes in either (**A**) various organs and/or developmental stages or (**B**) during seed development (i.e., 90-to-180 days-after-pollination [DAP]). Data presented are based on surveys of the tung transcriptome database [3]. Expression (i.e., transcript) levels are presented as normalized, log_10_-transformed FPKM (Fragments Per Kilobase Million) values and were formatted into heat maps using GraphPad Prism. Scales shown at the bottom of (**A**,**B**) indicate the correlation between colour-intensity and relative levels of gene expression; crossed boxes represent no detectable gene expression. Refer to Figure S3 for the co-expression analysis of the dataset presented in (**B**).

Since LDAPs are known to be important for packaging oil in both seeds and vegetative tissues in Arabidopsis [21,22,35], we utilized the tung transcriptome database [3] to survey the relative expression levels of the *VfLDAP* genes in tung. As shown in Figure 3A, all of the *VfLDAP*s are broadly expressed in a variety of organs and developmental stages, including young and mature leaves, stems, flowers, as well as developing seeds and roots, although the relative expression level of *VfLDAP1* is generally higher than that of *VfLDAP2* and *VfLDAP3* overall. In line with these results, several other tung genes known to be involved in oil synthesis and TAG compartmentation are also ubiquitously expressed in tung, such as those encoding the TAG-ESA biosynthetic enzymes VfDGAT2 [12], VfGPAT9 (GLYCEROL-3-PHOSPHATE ACYLTRANSFERASE 9) [36,37] and VfLPAT2 (LYSOPHOSPHATIDYL ACYLTRANSFERASE 2) [38], as well as the LD biogenetic proteins VfSEIPIN1 and 2 [39] and VfLDIP (LDAP-INTERACTING PROTEIN) [40] (Figure 3A). By contrast, the genes encoding VfFADX [13] and VfLDPS (LD-PROTEIN-OF-SEEDS) [41] are expressed in a more seed-specific manner (Figure 3A), which is similar to the expression of the tung *OLEOSIN* genes reported elsewhere [42].

Given that all three *VfLDAPs* are expressed in immature seeds (Figure 2A), their expression patterns, along with various other TAG biosynthetic and LD biogenetic genes, were more closely examined throughout seed development and maturation (Figure 3B). In tung seeds, ESA and oil rapidly accumulate during the latter stages of seed development and maturation, reaching their highest levels after approximately 130 days-after-pollination (DAP) and remaining high at 150 and 180 DAP [3], which is consistent with the pronounced upregulation of *VfFADX* expression during this same time course (Figure 3B; [3,43]). In contrast to *VfFADX*, *VfLDAP1-3* expression was highest after the plateaued accumulation of ESA and oil, with all three isoforms exhibiting their respective peaks at 180 DAP with *VfLDAP3* being the most highly expressed at this stage (Figure 3B). Overall, *VfLDAP* expression increased throughout seed maturation, with *VfLDAP1* showing the most consistent increase throughout seed development and maturation, while *VfLDAP2* and *VfLDAP3* exhibited their peak relative expression at the final measured stage (Figure 3B). In addition, co-expression analysis indicated that *VfLDAP1* and *VfLDAP2* expression during tung seed development and maturation positively correlated with the majority of other TAG-ESA and LD biogenetic genes examined, including *VfFADX*, *VfDGAT2*, *VfSEIPIN1* and *VfLDPS* (Figure S3). By contrast, *VfLDAP3* expression did not correlate with these other genes, nor with *VfLDAP1* or *VfLDAP2*, and instead was most highly expressed uniquely towards the end of seed development (Figure 3B and Figure S3). This may be indicative of differences in gene regulation and/or possible different functional roles for the various VfLDAP isoforms.

### 2.3. VfLDAP1, 2, and 3 Localize to LDs and Influence Their Numbers and Sizes When Transiently Expressed in N. benthamiana Leaves 

**Figure 4:**
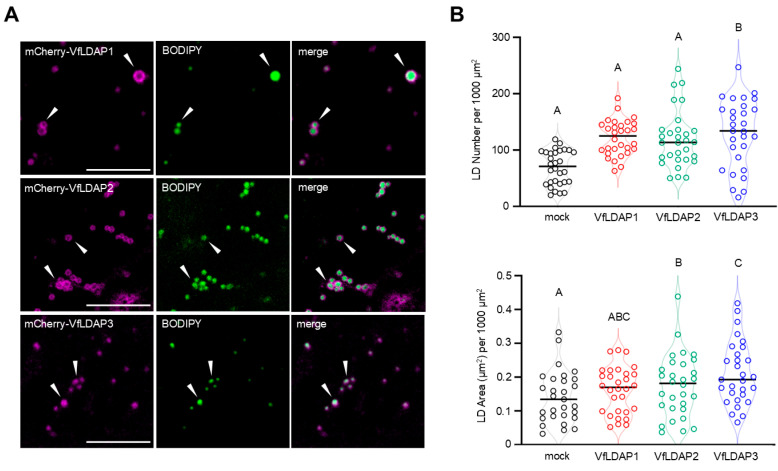
Transiently-expressed *VfLDAP*s in *N. benthamiana* leaves localize to LDs and alter LD size and abundance. (**A**) Representative CLSM micrographs (z-sections) of *N. benthamiana* leaf cells transiently expressing, as indicated by labels, mCherry-appended VfLDAP1-3 (false-coloured magenta) and stained with the neutral lipid-selective dye BODIPY 493/503. Shown also are the corresponding merged images. Arrowheads indicated examples of colocalization of the expressed fusion protein and LDs; note, in some instances, the torus-shaped fluorescence pattern attributable to the mCherry-VfLDAP surrounding a BODIPY-stained LD, indicating that the VfLDAPs are localized to the LD surface. Scale bars = 10 µm. (**B**) Quantification of LD abundance and sizes in *N. benthamiana* leaf cells transiently expressing individual (native) *VfLDAP*s or *mCherry* alone (referred to here as ‘mock’), as indicated with labels. Identification of cells expressing the *VfLDAP*s was based on the fluorescence attributable to co-expressed *mCherry*, which served as marker for cell transformation; refer also to Figure S4 for RT-PCR analysis results confirming the expression of each *VfLDAP*, as well as *VfFADX* and *VfDGAT2* in *N. benthamiana* leaves. LDs were stained with BODIPY and LD numbers and areas were measured using ImageJ. Values shown in violin plots represent those obtained from three biological replicates (i.e., three separate plant infiltration experiments), with each replicate consisting of 15 micrographs (z-stacks of 1000 μm^2^) obtained from two different infiltrated leaves. The horizontal line shown for each plot indicates the median value. Letters represent statistically significant differences of at least *p* ≤ 0.05 relative to the mock, as determined by Mann–Whitney U tests.

We next analyzed the subcellular localization of VfLDAPs and their potential impact on LD numbers and sizes when transiently expressed in *N. benthamiana* leaves, which is a well-established model system for studying plant protein targeting and organelle biogenesis [44]. More specifically, individual *VfLDAPs* either with or without an N-terminal-appended mCherry fluorescent protein were transiently expressed (via *Agrobacterium tumefaciens*-mediated infiltration) in *N. benthamiana* leaves, then LDs were stained with the neutral lipid-specific dye boron-dipyrromethene (BODIPY) [45] and visualized using confocal laser-scanning microscopy (CLSM). As shown in Figure 4A, all three VfLDAP-mCherry fusion proteins localized specifically to LDs and often yielded a distinct torus fluorescence pattern, indicative of their localization to the surface of LDs and consistent with the localization of other LD coat proteins ectopically expressed in plant cells [46]. 

We also observed that the transient expression of *VfLDAPs* lacking an appended mCherry tag in *N. benthamiana* leaves resulted in a slight increase in the number of LDs per cell relative to mock-transformed leaves (i.e., leaves transformed with only the viral RNA-silencing suppressor P19), although only VfLDAP3 yielded a significant change (Figure 4B). Similarly, the transient expression of the individual *VfLDAPs* in leaf cells also resulted in an increase in LD size, although only *VfLDAP2* and *VfLDAP3* were statistically significant different in comparison to the mock (Figure 4B). These data confirm that all three of the putative VfLDAPs localize to LDs and, based on their ability to influence LD numbers and sizes, play a role(s) in LD dynamics in plant cells.

### 2.4. VfLDAPs Transiently Expressed in N. benthamiana Leaves Differentially Affect TAG Content and Fatty Acid Composition

**Figure 5:**
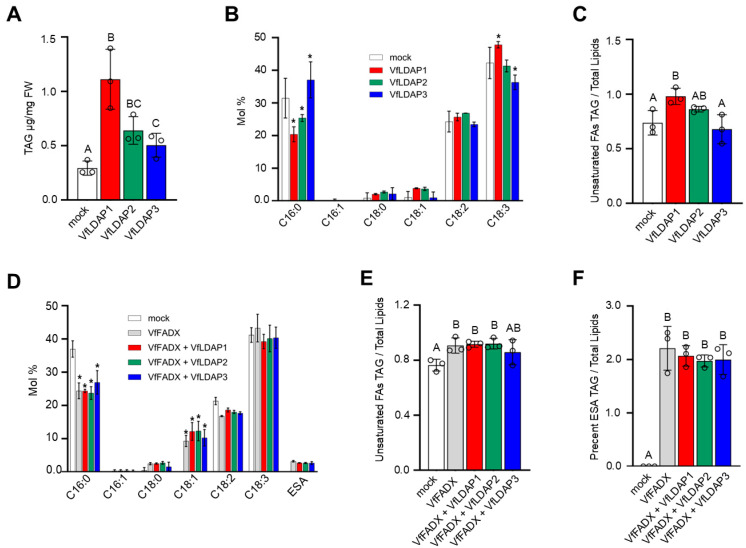
Effects of *VfLDAP* and/or *VfFADX* ectopic expression on TAG content and fatty acid composition in *N. benthamiana* leaves. Total lipids were extracted from leaves transiently (co)expressing either *VfLDAP1-3* on their own or with *VfFADX*, *VfFADX* on its own, or only the viral suppressor *P19* (i.e., referred to as ‘mock’), as indicated with labels. Total lipids were then analyzed by TLC and GC-FID for (**A**) total TAG content (μg/mg fresh weight [FW]), (**B**,**D**) fatty acid composition of TAG, (**C**,**E**) proportion of unsaturated fatty acids in TAG compared to total lipids, and (**F**) percentage of ESA in TAG compared to total lipids; refer to Section 4 for additional details. Values shown represent the means ± standard deviation (SD) from three biological replicates (i.e., leaf materials harvested from three separate plant infiltrations). Asterisks in (**B**,**D**) represent statistically significant differences of at least *p* ≤ 0.05 related to the mock, as determined by a two-way Analysis of Variance (ANOVA) followed by a Tukey’s post-hoc multiple comparison test. Letters in (**A**,**C**,**E**,**F**) represent statistically significant differences of at least *p* ≤ 0.05 relative to the mock, as determined by Welsh’s *t*-tests.

Given their ability to influence LD abundance and size (Figure 4B), we next analyzed whether transient expression of the *VfLDAPs* in leaves also affected the content and/or composition of TAG, which is the principal neutral lipid stored in the LDs of most plant cells [19,47]. As shown in Figure 5A, transient expression of *VfLDAP1*, *2*, or *3* resulted in significant increases in TAG content in *N. benthamiana* leaves in comparison to the mock control, with VfLDAP1 yielding the greatest relative increase in TAG abundance. Transient expression of the *VfLDAPs* also had variable effects on the fatty acid composition of TAG (Figure 5B). Expression of *VfLDAP1* resulted in a decrease in palmitic acid (C16:0) content and, simultaneously, an increase in linolenic acid (C18:3Δ^9*cis*,12*cis*,15*cis*^) content. Similarly, a slight decrease in palmitic acid content was also observed with expression of VfLDAP2 (Figure 5B). By contrast, expression of *VfLDAP3* yielded an inverse trend in the fatty acid composition of TAG with an increase in palmitic acid and decrease in linolenic acid (Figure 5B). In fact, overall, there was a slightly greater proportion of unsaturated fatty acids in the TAG fraction of leaves transformed with *VfLDAP1* compared to the mock control (Figure 5C).

Since tung oil contains high amounts of TAG-ESA [3,43], we examined next whether the VfLDAPs might influence the accumulation of α-ESA-containing TAGs (referred to henceforth as TAG-ESA) by co-expressing each of the individual *VfLDAPs* with *VfFADX* in *N. benthamiana* leaves. As mentioned, VfFADX is a divergent form of FAD2 responsible for the synthesis of ESA in tung seeds [13]. Expression of *VfFADX* alone in *N. benthamiana* leaves resulted in a significant increase in TAG content (Figure S5A) and changes in TAG fatty acid composition, including a decrease in palmitic acid, increase in oleic acid (C18:1Δ^9*cis*^), and appearance of ESA (Figure 5D). Also observed in leaves expressing *VfFADX* on its own was a significant increase in the ratio of unsaturated fatty acids in TAG versus total lipids (Figure 5E), suggesting that the production of ESA by VfFADX stimulates the steady-state accumulation of unsaturated fatty acids in TAG, in general. Co-expression of *VfFADX* with each of the individual *VfLDAPs*, however, did not lead to any additional increases in TAG content and/or changes in fatty acid composition, nor changes in the ratio of unsaturated fatty acids in TAG or the accumulation of ESA versus total lipids in comparison to expression of *VfFADX* alone (Figure 5D,F).

### 2.5. Transient Co-Expression of VfFADX, VfDGAT2, and VfLDAP2 or VfLDAP3 in N. benthamiana Leaves Results in an Increase in ESA Content in LDs

**Figure 6:**
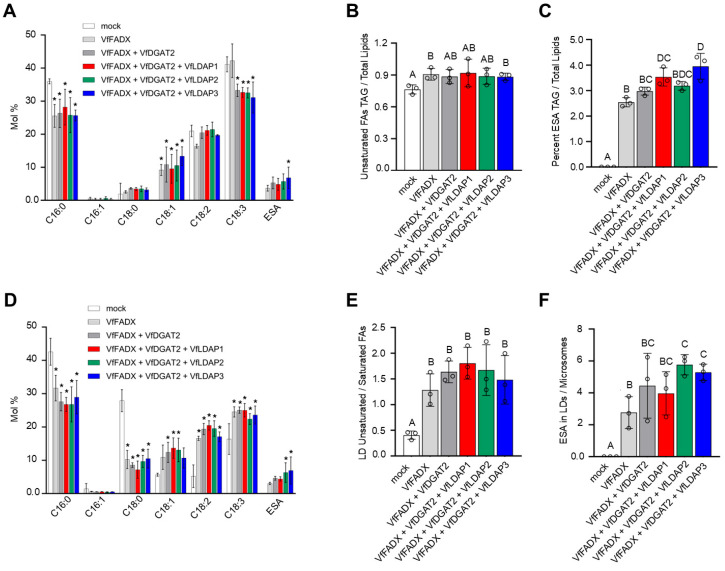
Effects of *VfLDAP*, *VfFADX* and/or *VfDGAT2* ectopic expression on TAG content and fatty acid composition, as well as distribution of ESA between LDs and microsomes isolated from *N. benthamiana* leaves. Total lipids were extracted from either (**A**–**C**) leaves or (**D**–**F**) LDs and ER microsomal fractions isolated from leaves transiently (co)expressing either *VfFADX* on its own or with *VfDGAT2* and/or *VfLDAP1-3*, or *P19* (i.e., ‘mock’), as indicated with labels. Total lipids were then analyzed by TLC and/or GC-FID for (**A**,**D**) fatty acid composition of TAG, (**B**) proportion of unsaturated fatty acids in TAG compared to total lipids, (**C**) percentage of ESA in TAG compared to total lipids; (**E**) proportion of unsaturated versus saturated fatty acids in LDs and (**F**) proportion of ESA in isolated LDs compared to ER microsomes; refer to Section 4 for additional details. Values shown represent the means ± SD from three biological replicates from three separate plant infiltrations. Asterisks in (**A**,**D**) represent statistically significant differences of at least *p* ≤ 0.05 related to the mock, as determined by a two-way ANOVA followed by a Tukey’s post-hoc multiple comparison test. Letters in (**B**,**C**,**E**,**F**) represent statistically significant differences of at least *p* ≤ 0.05 relative to the mock, as determined by Welsh’s *t*-tests.

To more fully explore the function of VfLDAPs in packaging TAGs containing ESA, each of the individual *VfLDAPs* was co-expressed with both *VfFADX* and *VfDGAT2*, the latter of which is responsible for the incorporation of ESA into the *sn*-3 position of TAG [12,14]. While the co-expression of *VfDGAT2* with *VfFADX* did not result in a significant increase in TAG abundance compared to when *VfFADX* was expressed on its own (Figure S5B), there were significant changes in the fatty acid composition of TAG (Figure 6A). More specifically, compared to *VfFADX* expressed alone, co-expression of *VfFADX* and *VfDGAT2* resulted in a decrease in linolenic acid and modest increase in ESA content, although not statistically significant (Figure 6A). Further, although the ratio of unsaturated fatty acids in TAG versus total lipids remained the same (Figure 6B), the ratio of ESA in TAG versus total lipids slightly increased upon co-expression of *VfFADX* and *VfDGAT2* compared to *VfFADX* expressed alone (Figure 6C), which supports the role of VfDGAT2 in improving channeling of ESA into TAG [12,14]. Further, co-expression of *VfLDAP1*, *2*, or *3* with both *VfFADX* and *VfDGAT2* only led to minimal changes in TAG composition. Co-expression of *VfLDAP3* with both *VfFADX* and *VfDGAT2* specifically led to a slight, but significant increase in the proportion of ESA in TAG compared to when only *VfFADX* and *VfDGAT2* were co-expressed (Figure 6A). By contrast, none of the combinations co-expressing *VfLDAP1* or *VfLDAP2* affected TAG content, fatty acid composition, or the proportion of unsaturated fatty acids in TAG versus total lipids (Figure 6A,B and Figure S4B). Furthermore, co-expression of *VfLDAP3*, but not *VfLDAP1* or *VfLDAP2*, with *VfFADX* and *VfDGAT2* resulted in a significant increase in ESA in TAG relative to total lipids in comparison to leaves co-expressing *VfFADX* and *VfDGAT2* (Figure 6C).

We next examined whether the VfLDAPs are potentially involved in the partitioning of TAG from the ER into LDs. To address this possibility, LDs and ER microsomal fractions were isolated from infiltrated *N. benthamiana* leaves and subjected to lipid analysis. As shown in Figure 6D, lipids (majority TAG) extracted from the isolated LDs of leaves co-expressing *VfFADX* and/or *VfDGAT2*, along with each of the *VfLDAPs*, displayed some differences in their fatty acid profiles compared to those observed in the corresponding whole leaf TAG fractions (see Figure 6A). For example, there were significant changes in overall fatty acid composition of LDs isolated from leaves expressing *VfFADX* and/or *VfDGAT2* relative to the mock control, including increases in linoleic acid, linolenic acid, and ESA content, as well as decreases in palmitic and stearic acids (Figure 6D). Furthermore, co-expression of *VfLDAP2* or *VfLDAP3* with *VfFADX* and *VfDGAT2* resulted in a significant increase of ESA content in isolated LDs (Figure 6D), and this increase was specific for ESA and not for unsaturated fatty acids, in general (Figure 6E). Further, the ratio of ESA in LDs versus ER microsomes was also statistically increased by co-expression of *VfLDAP2* or *VfLDAP3* (Figure 6F), suggesting that the corresponding two proteins promote enrichment of TAG-ESA in LDs. Taken together, these results suggest that the VfLDAPs may have differences in their ability to enrich TAGs with different fatty acid compositions.

## 3. Discussion

### 3.1. Identification and Characterization of Three LDAP Isoforms (VfLDAP1-3) in Tung

Like most other plant species, the tung tree contains three distinct LDAP isoforms (VfLDAP1-3) that are similar, both in terms of their sequences and structural features, as well as with their respective isoforms from other plants. This indicates a deep evolutionary conservation of the LDAP three-gene family (Figure 1, Figure S1 and Figure S2). While the LDAPs lack any known enzymatic functions, they contain a number of conserved elements (Figure 2), including a hydrophobic region near the N-terminus that might be important for association with the LD surface. LDAPs also contain a conserved REF domain of unknown function that is shared with the SMALL RUBBER PARTICLE PROTEINs (SRPPs) of rubber-accumulating plant species [48,49,50]. Interestingly, LDAPs and SRPPs show similarities in polypeptide sequence [22] but are involved in packaging different types of lipids into LDs, with the former packaging mainly TAGs, and the latter packaging polyisoprenes [50,51]. These observations suggest that LDAPs in general may have evolved the capacity to stabilize LDs containing different neutral lipid compositions.

The functional specialization of LDAPs is also supported by previous experiments in Arabidopsis showing that the three *AtLDAP* isoforms are differentially expressed in various tissues and developmental stages, as well as induced during different abiotic stress responses [21,35]. Similarly, an evaluation of *VfLDAP1-3* gene expression in tung revealed some differential expression patterns in various organs and developmental stages (Figure 3A). Notably, all three *VfLDAP* genes are expressed during seed development and maturation, with *VfLDAP1* being more consistently expressed over time, while *VfLDAP2* and *VfLDAP3* show their highest relative expression towards the end of seed development (Figure 3B), suggesting that the isoforms might function in different ways during seed development and/or seed oil accumulation.

Transient expression of the *VfLDAPs* in *N. benthamiana* leaves confirmed that each of the protein isoforms targeted specifically to LDs (Figure 4A), as observed previously for studies of LDAPs from Arabidopsis [21,35] and pennycress (*Thlaspi arvense* L.) [34]. Similar also to prior studies with the Arabidopsis and pennycress LDAPs [21,34], transient expression of the *VfLDAPs* in *N. benthamiana* leaves resulted in a significant increase in the number of LDs and a modest increase in LD size (Figure 4B). Although the underlying mechanisms of these changes are currently unknown, the increase in number and size of LDs upon *VfLDAP* over-expression supports their proposed role(s) in LD biogenesis in plants in general [52], as changes in LD morphology (size or number) have been reported following the overexpression and/or knockout of other LD coat protein types [19]. For instance, overexpression of the *OLEOSINs*, which are considered to function primarily as structural LD coat proteins, results in smaller and more numerous LDs [19,21,25,26], while disruption in the expression of *OIL BODY ASSOCIATED PROTEIN 1*, which is also considered a structural LD coat protein and has also been proposed to be a transcription factor interactor, yields LDs of larger size and numbers [53,54]. Similarly, a loss of *CALEOSIN 1*, which is also a structural LD coat protein with calcium-binding and peroxygenase activity, results in an increase in LD number [55]. Notably, alternations in the expression of LD coat proteins are also often accompanied by increases and/or changes in cellular neutral lipid content and composition [19,21,25,26,53,55].

### 3.2. Transient Expression of Tung LDAP Isoforms Differentially Alters TAG Content and Composition in Leaves and VfLDAP2 and VfLDAP3 Enrich ESA in a Partially Reconstituted TAG-ESA Biosynthetic Pathway

Analysis of lipids from *N. benthamiana* leaves transiently expressing each of the *VfLDAPs* revealed increases in TAG content relative to the mock control (Figure 5A), which was somewhat expected, since the number and size of LDs was increased (Figure 4B). However, the amount of TAG that accumulated in leaves varied between the three VfLDAPs, with the highest amount observed with VfLDAP1 (Figure 5A). The VfLDAPs also had differential effects on TAG fatty acid composition, with the largest changes observed for palmitic and linolenic fatty acids (Figure 5B). Collectively, these results demonstrate that the VfLDAPs have different capacities to influence the steady-state content and composition of neutral lipids in a transgenic system (i.e., *N. benthamiana* leaves). Co-expression of each *VfLDAP* with *VfFADX* and *VfDGAT2* further showed that VfLDAP3 significantly increased the percentage of ESA in total leaf TAG (Figure 6A), and both *VfLDAP2* and *VfLDAP3* co-expressed with *VfFADX* and *VfDGAT2* increased relative ESA abundance within isolated LDs (Figure 6D). This increase of ESA was possibly due to VfLDAP2- or VfLDAP3-dependent partitioning of TAG-ESA from the ER into LDs, as reflected by the increased ratio of ESA in isolated LDs versus ER microsomes in leaves (Figure 6F). However, it is also possible that VfLDAP2 and VfLDAP3 differentially influence access of the LD TAG core to lipases or other TAG remodeling enzymes, which are known to be active during the latter stages of seed development [43,56,57]. Distinguishing between these and possibly other mechanisms will require future investigation.

### 3.3. Potential Mechanisms by Which LD Coat Proteins Modulate TAG Content and Composition in LDs

Recent experimental studies, including molecular dynamics simulations, have revealed that the LD phospholipid monolayer exhibits transient “packing defects” that expose the underlying neutral lipid core (as reviewed in [58,59]). This is thermodynamically unfavorable and creates the potential for the binding of LD surface-associated proteins. Indeed, numerous, so-called type-II LD coat proteins, which target to the LD surface directly from the cytoplasm [56], utilize the hydrophobic face of amphipathic α-helices to bind to the LD surface, with large hydrophobic amino acid side chains filling the pockets generated by the membrane packing defects [58,60]. The local physicochemical properties of these membrane packing defects are likely to be influenced, at least in part, by the composition of the neutral lipid core. For instance, a neutral lipid core composed of TAGs enriched in monounsaturated and saturated fatty acids might result in a certain biophysical and thermodynamic microenvironment that is different than a neutral lipid core composed of TAGs containing polyunsaturated fatty acids. It is possible, therefore, that LD coat proteins have evolved to bind more efficiently and effectively to LDs containing different compositions within the neutral lipid core. The stabilization of the LD membrane surface could help protect the neutral lipid core from TAG metabolizing enzymes, such as lipases and/or acyltransferases, thereby influencing the steady state content and composition of TAG.

In a recent study of *Physaria fendleri*, which produces a seed oil containing high amounts of hydroxylated fatty acids, it was shown that TAG remodeling towards the latter stages of seed development plays an important role in enrichment of the unusual fatty acid in seed oil [57]. Here, we showed that the relative levels of *VfLDAP2* and *VfLDAP3* gene expression are highest towards the end of tung seed development (Figure 3B), and that both proteins show a capacity for increasing the accumulation of ESA in neutral lipids, primarily within LDs, in a partially reconstituted TAG-ESA biosynthetic system (i.e., *VfFADX* and *VfDGAT2* co-expression) (Figure 6F). Taken together, these observations suggest that VfLDAP2 and/or VfLDAP3 might be involved in the selective packaging and stabilization of LDs containing elevated amounts of TAG-ESA, possibly by binding to membrane packing defects that expose a neutral lipid core enriched in TAG-ESA and/or associated protein interactors. Notably, there is increasing evidence that other LD coat proteins, particularly the PLIN proteins in mammals, are associated with distinct LD subpopulations with different neutral lipid cores and interact with a diverse array of interactors [61,62,63,64]. Whether such diversification of LDs and/or LD protein interactions exist in plants remains to be determined. Regardless, our results here with the VfLDAPs, as well as those from other studies involving the ectopic expression of OLEOSINs in transgenic plants [30,31,33,65,66], suggest that LD coat proteins are important tools for increasing oil content in plants and perhaps tailoring fatty acid composition for specific purposes.

## 4. Materials and Methods

### 4.1. Sequence, Phylogenetic, and Structural Analyses of VfLDAPs

Tung LDAP homologs were identified through a series of pBLAST searches of the tung transcriptome (National Center for Biotechnology Information [NCBI] Genome Project No: PRJNA770124; [3]) using the Arabidopsis *LDAP1*, *2*, and *3* open reading frames (ORFs) as queries. The resulting deduced amino acid sequences were analyzed using the online tools: Clustal Omega available on EMBL-EBI (https://www.ebi.ac.uk/; [67]; accessed on 10 July 2024), Transmembrane Helices Hidden Markov Model (TMHMM v.2.0; http://www.cbs.dtu.dk/services/TMHMM/; [68]; accessed on 25 July 2024), and the InterPro database Motif search tool [69] (accessed on 25 July 2024), using default parameters. To identify LDAPs from other plant species, VfLDAP- and AtLDAP-deduced amino acid sequences were individually queried against the deduced proteomes of all angiosperm plant species available at Phytozome (https://phytozome-next.jgi.doe.gov/; [70]; accessed on 1 August 2024). Putative LDAPs were then subjected to 100-replicate multiple sequence alignments using the MUSCLE algorithm implemented in MEGA-X (v.11.0.9; https://www.megasoftware.net/; [71]; accessed on 30 July 2024). Resulting alignments were used to generate a consensus maximum-likelihood tree (Poisson model) with bootstrap values presented as circles (individual sizes proportional to bootstrap values) and a sequence-identity matrix, which was visualized with iTOL (v.6; https://itol.embl.de/ [72]; accessed on 4 August 2024) and GraphPad Prism (v.10.3.1; https://www.graphpad.com/), respectively. Similarly, other tung TAG-ESA biosynthetic and LD biogenesis-associated homologs were identified using pBLAST searches of the tung transcriptome with corresponding Arabidopsis genes as queries; refer below to the ‘Gene Accession Numbers’ section for additional details on Arabidopsis genes used as queries.

The structural models of VfLDAPs and other LDAP isoforms were generated using the default open access AlphaFold Protein Structure Database (https://alphafold.com/; [73]; accessed in 30 July 2024) and visualized with PyMOL (v. 2.5.7; [74]).

### 4.2. Plasmid Construction

The ORFs of VfLDAP1-3 were custom synthesized (Integrated DNA Technologies; Coralville, USA) and used as templates in polymerase chain reactions (PCR) with gene-specific primers that included flanking 5′ and 3′ attB sites; all primers used in this study for cloning or reverse transcriptase (RT)-PCRs (see below) are listed in Table S2. Cycling conditions for PCR-based cloning consisted of 35 cycles of 95 °C for 30 s, 52 °C for 30 s, and 72 °C for 60 s. The resulting PCR products were subcloned into pDONR Zero^TM^ Gateway vector [75] and then into the Gateway-compatible, plant binary expression vectors, pMDC32 [75] and pMDC32/N-mCh, the latter of which encodes the red fluorescent protein, mCherry, followed by an in-frame multiple cloning site [76]. Both binary vectors contain the constitutive 2 *× 35S Cauliflower Mosaic Virus* promoter. The construction of other binary vectors, including *pMDC32/VfFADX* and *pMDC32/VfDGAT2-B9*, encoding VfFADX and VfDGAT2, respectively, and *pORE04/P19*, encoding the Tomato bushy stunt virus (TBSV) RNA-silencing suppressor P19, have been described elsewhere [14,52]. All new plasmid constructs were confirmed by automated sequencing at the University of Guelph Genomics Facility.

### 4.3. Transient Expression in N. benthamiana Leaves

*N. benthamiana* plants used for transient transformation experiments were grown in soil in a growth chamber at 22 °C with a 16-h-day/8-h-night cycle and 200 μE m*^−^*^2^ s*^−^*^1^ light intensity. Leaves of ~28-day-old *N. benthamiana* plants were infiltrated with transgenic *A. tumefaciens* (strain LBA4404) carrying appropriate binary vectors, as previously described [21,52]. *A. tumefaciens* transformed with TBSV *P19* was also included in all infiltrations to enhance transgene expression [77]. Details on the transformation of *A. tumefaciens*, as well as those related to the growth, transformation, infiltration, and processing of *N. benthamiana* leaf materials for microscopy, have been described elsewhere [21,39,52].

### 4.4. RT-PCR

Confirmation of expression of transgenes in *N. benthamiana* leaves, 3 days post-infiltration (DPI), was carried out using RT-PCRs (Figure S4), based on procedures described previously [21,39]. Briefly, RNA was extracted from ~100 mg of infiltrated *N. benthamiana* leaf tissue, flash-frozen in liquid nitrogen, and then manually ground into a fine powder using plastic pellet pestles. RNA was then isolated using the RNeasy Plant Mini Kit (Qiagen; Toronto, ON, Canada) and used as a template for the synthesis of complementary DNA (cDNA) with the QuantiTect Reverse Transcription Kit (Qiagen; Toronto, ON, Canada). Genomic DNA was isolated from *N. benthamiana* leaves according to the method outlined in [76]. *N. benthamiana* ACTIN served as a reference gene for all RT-PCRs and cycling conditions consisted of 35 cycles of 95 °C for 30 s, 52 °C for 30 s, and 72 °C for 60 s. All gene-specific primers used for RT-PCRs are listed in Table S2.

### 4.5. Confocal Microscopy

*N. benthamaina Agrobacterium*-infiltrated leaves (3 DPI and ~1.5 h following the end of the night cycle when LD abundance was relatively high [21]) were processed, fixed in formaldehyde, and LDs stained with BODIPY 493/503 (Invitrogen; Burlington, ON, Canada), as previously described [52]. Micrographs were acquired with a Leica SP5 CLSM equipped with a 63× glycerol-immersion objective (NA = 1.3), and five laser systems, including an Arion laser, green, orange, and red HeNe lasers, and a Radius 405-nm laser (Leica Microsystems, Concord, ON, Canada). *N. benthamiana* leaf cell micrographs were captured as single optical sections (i.e., z-sections) or z-stacks (consisting of 0.4 μm z-sections, 15 μm in total) and saved as 512 *×* 512-pixel images. Excitations and emission signals for fluorescent proteins and neutral lipid-specific dyes collected sequentially in double- or triple-labeling experiments were the same as those described previously [21,39]; single-labeling experiments showed no detectable crossover at the settings used for data collection.

All fluorescence images of plant cells shown in individual figures are representative of at least three separate experiments. LDs were quantified from z-stack micrographs captured as previously described and as in [39], using the number and area outputs tabulated by the “Analyze Particles” function (at default settings, with the exception of a circularity value of 0.90–1.0) in ImageJ (v.1.43; https://imagej.net/ij/; [78]; accessed on 30 July 2024). All figure compositions shown in the paper were generated and images therein processed for brightness and contrast using Microsoft^®^ PowerPoint (v.16.76.1).

### 4.6. LD and ER Microsomal Isolations

LDs and ER microsomes were isolated from infiltrated *N. benthamiana* leaves using methods described in [79]. Briefly, ~2.5 g of infiltrated leaves was harvested at 3 DPI and homogenized on ice with 600 mM sucrose buffer (600 mM sucrose, NaH_2_PO_4_, pH 7.5, 150 mM NaCl, 0.1% [*v*/*v*] Tween-20, 1 mM PMSF, 1× Complete™ Protease Inhibitor Cocktail [Roche Canada; Mississauga, ON, Canada]). The resulting suspension was transferred to 15 mL glass centrifuge tubes on ice and 2.5 mL of ice-cold 400 mM sucrose buffer (400 mM sucrose, 10 mM NaH_2_PO_4_, pH 7.5, 150 mM NaCl, 0.1% [*v*/*v*] Tween-20, 1 mM PMSF, 1× Complete™ Protease Inhibitor Cocktail was then layered on top of the leaf homogenate to create two phases. The samples were then centrifuged at 10,500× *g* for 60 min at 4 °C to separate LDs by flotation. LDs were collected from the top of the uppermost sucrose buffer for subsequent lipid analysis. The remaining supernatant was transferred to ultracentrifuge tubes and further centrifuged at 100,000× *g* for 60 min at 4 °C. The resultant pellet, which consists primarily of ER microsomes, was then resuspended in the 400 mM sucrose buffer before proceeding to lipid analysis.

### 4.7. Lipid Analysis

For qualitative analysis of lipids from infiltrated *N. benthamiana* leaves, total lipids were extracted from ~700 mg of fresh leaf tissues, 3 DPI, using modified methods from [14,80]. Briefly, fresh tissue was weighed, snap-frozen in liquid nitrogen, manually ground into a powder and thoroughly vortexed in an extraction solvent composed of methanol:chloroform:formic acid (20:10:1 [*v*/*v*/*v*]), and 0.01% (*v*/*v*) butylated hydroxy toluene (BHT) for 5 min. C17:0 TAG (Sigma-Aldrich; Oakville, ON, Canada) was added as an internal standard. The total lipids in the chloroform phase were collected, dried with inert nitrogen gas, and resuspended in chloroform containing 0.01% (*v*/*v*) BHT. To separate the TAG fraction, total lipids were run on a silica thin-layer chromatography (TLC) plate using hexane:diethyl ether:acetic acid (70:30:1 [*v*/*v*/*v*]) as the mobile phase and stained with 0.05% (*v*/*v*) primulin dissolved in 80% (*v*/*v*) acetone for visualization under ultraviolet light [14]. For analysis of lipids from isolated LDs and ER microsomes, total lipids were extracted from LD and ER microsomal preparations using methanol:chloroform:formic acid (20:10:1 [*v*/*v*/*v*]) as described above.

Isolated lipids were subjected to transmethylation using sodium methoxide, as described previously [14]. The resulting fatty acid methyl esters (FAMEs) were extracted and resuspended in hexane containing 0.01% (*v*/*v*) BHT and then analyzed by gas chromatography with a flame ionization detector (GC-FID) using an Agilent 8890 system equipped with an Agilent DB-23 (Agilent, Santa Clara, CA, USA) capillary column (30 m × 0.25 mm × 0.25 μm). Samples were run with the following method: hold at 165 °C for 4 min, ramp from 165 °C to 180 °C at 10 °C/min, hold at 180 °C for 3 min, ramp from 180 °C to 190 °C at 10 °C/min, ramp from 190 °C to 198 °C at 1 °C/min, ramp from 198 °C to 230 °C at 15 °C/min, and hold at 230 °C for 2 min. FAME peaks were identified by comparison with the CRM18918 (C8–C24) standard F.A.M.E. mix (Sigma-Aldrich) and FAMEs prepared from commercially available tung oil (Home Depot^®^ Canada, Guelph, ON, Canada). Note that all ESA quantification results presented in this study refer specifically to α-eleostearic acid (i.e., α-ESA), rather than its naturally less abundant isomer, β-eleostearic acid (i.e., C18:3Δ^9*trans*,11*trans*,13*trans*^; β-ESA) [13], or their combination; refer to Figure S6 for confirmation of GC-FID-based separation of α-ESA and β-ESA.

### 4.8. Statistical Analysis

Statistical significance of LD number and size was determined with a series of pairwise Mann–Whitney U test’s (*p* ≤ 0.05). Statistical analyzes of fatty acid compositions of TAG, isolated LDs and microsomal fractions were carried out with two-way ANOVA tests, followed by Tukey’s post-hoc multiple comparison tests and TAG quantification analyzes were assessed with pairwise Welsh’s *t*-tests. Variance homogeneity was confirmed for above lipid-specific datasets with a series of Levene’s tests. Gene co-expression was analyzed with Pearson’s correlation tests followed by Student’s t-tests. All statistical tests were conducted using GraphPad Prism. Statistical analyses used for phylogenetic tree constructions were performed using MEGA-X.

### 4.9. Gene Accession Numbers

Tung tree transcriptional data can be accessed from the Genome Sequence Archive at the Beijing Institute of Genomics (BIG) Data Center, Chinese Academy of Sciences (GSA: CRA001732), and are publicly accessible at https://bigd.big.ac.cn/gsa/, accessed on 30 July 2024. Accession numbers for tung genes examined in this study are as follows: *VfLDAP1* (tung.gene.scaffold86.00052), *VfLDAP2* (tung.gene.scaffold608.00013), *VfLDAP3* (tung.mrna.scaffold251.00189), *VfFADX* (tung.gene.scaffold1603.00001), *VfDGAT2* (tung.gene.scaffold2251.00002), *VfLPAT2* (tung.gene.scaffold1708.00007), *VfGPAT9* (tung.gene.scaffold621.00001), *VfSEIPIN1* (tung.gene.scaffold76.00075); *VfSEIPIN2* (tung.gene.scaffold679.00031), *VfLDPS* (tung.gene.scaffold101.00003), and *VfLDIP* (tung.gene.scaffold2314.00002). Accession numbers of other genes described in this study include *N. benthamiana ACTIN* (NCBI AY179605.1) and Arabidopsis *LDAP1* (Arabidopsis gene identifier [AGI] locus AT1G67360), *LDAP2* (AT2G47780), *LDAP3* (AT5G16550), *AtDGAT2* (AT2G19450), *AtLPAT2* (AT3G57650), *AtGPAT9* (AT5G60620), *AtSEIPIN1* (AT5G16460), *AtSEIPIN2* (AT1G29760), *AtSEIPIN3* (AT2G34380), *AtLDPS* (AT3G19920), *AtLDIP* (AT5G16550).

## Data Availability

Data are contained within the article or Supplementary Material, and additional information is available on request from the authors.

## Supplementary Materials

The following supporting information can be downloaded at: www.mdpi.com/article/10.3390/plants14050814/s1, Table S1: List of VfLDAP and AtLDAP homologs used for phylogenetic analyses (Figure 1), AlphaFold-predicted protein structures (Figure 2 and Figure S2), as well as precent-deduced amino acid sequence identity matrix (Figure S1); Table S2: List of primers used in this study for RT-PCRs, molecular cloning and plasmid construction; Figure S1: Precent-deduced amino acid sequence identity matrix of top ten tung (*V. fordii*) and Arabidopsis (*A. thaliana*) protein homologs in Angiospermae; Figure S2: Comparison of AlphaFold-predicted three-dimensional structures of the tung (*V. fordii*), Arabidopsis (*A. thaliana*), castor bean (*Rinus communis*) and cassava (*Manihot esculenta*) LDAP protein isoforms; Figure S3: Heatmap depicting Pearson’s correlation coefficients and statistical significance for the co-expression of select tung genes across seed development (i.e., 90 to 180 days Days-After-Pollination [DAP]) in relation to *VfFADX*; Figure S4: Effects of VfFADX, VfDGAT2 and/or VfLDAP ectopic expression on TAG content in *N. benthamiana* leaves; Figure S5: Confirmation of transient gene expression in *N. benthamiana* leaves using RT-PCR. mRNA was isolated from *N. benthamiana* leaves 3 days-post-infiltration with the indicated constructs (i.e., *VfLDAP1-3*, *VfFADX* or *VfDGAT2*) or mock infiltrate (i.e., exclusively *P19*); Figure S6: Representative GC-FID chromatograms of methylated fatty acid (FAME) standards used in this study.

## Abbreviations

The following abbreviations are used in this manuscript:

LDAP	LIPID DROPLET-ASSOCIATED-PROTEIN
TAG	Triacylglycerol
LD	Lipid Droplet
ESA	Eleostearic Acid
FADX	FATTY ACID DESATURASE X
DGAT2	DIACYLGLYCEROL ACYLTRANSFERASE 2
ER	Endoplasmic Reticulum
DAP	Days Post Pollination
LDPS	LIPID DROPLET PROTEIN OF SEEDS
REF	Rubber Elongation Factor
FAD2	FATTY ACID DESATURASE 2
SRPP	SMALL RUBBER PARTICLE PROTEIN
PLIN	PERILIPIN
BLAST	Basic Local Alignment Search Tool
CLSM	Confocal Laser Scanning Microscopy
BODIPY	Boron-Dipyrromethene
LDIP	LIPID-DROPLET-INTERACTING-PROTEIN
GPAT9	GLYCEROL-3-PHOSPHATE ACYLTRANSFERASE 9
LPAT2	LYSOPHOSPHATIDYL ACYLTRANSFERASE 2
