# Supplementary material for: Identification and Characterization of Lipid Droplet-Associated Protein (LDAP) Isoforms from Tung Tree (Vernicia fordii)
Source: Plants (Basel). 2025 Mar 5;14(5):814. doi: 10.3390/plants14050814 (PMC11901875; doi:10.3390/plants14050814)
Supplement: Supplementary file 1 [file plants-14-00814-s001.zip › [FULL] Supplemental Figure Captions.pdf]

[FULL] Supplemental Figure Captions

Table S1. List of VfLDAP and AtLDAP homologs used for phylogenetic analyses (Figure 1), AlphaFold-predicted protein structures (Figure 2 and Figure S2), as well as precent-deduced amino acid sequence identity matrix (Figure S1). Included are the *Phytozome (https://phytozome-next.jgi.doe.gov/), °TAIR (https://www.arabidopsis.org/) and tung tree transcript accessions (Cui et al., 2018), as well as deduced amino acid sequences, species, and genus.

Table S2. List of primers used in this study for RT-PCRs, molecular cloning, and plasmid construction. All plasmid constructs used for subcloning and N. benthamiana infiltration are listed under "Cloning and plasmid construction primers" along with the cloning method and forward and reverse primer pairs used in their construction. Plant expression (binary) plasmids were generated using standard Gateway methods (i.e., BP and LR clonases). All transcripts amplified for RT-PCRs are listed under "RT-PCR primers" along with the forward and reverse primers used to amplify them.

Figure S1. Precent-deduced amino acid sequence identity matrix of top ten tung (V. fordii) and Arabidopsis (A. thaliana) protein homologs in Angiospermae. Homologs identified following individual tung and Arabidopsis LDAP pBLAST searches of the Phytozome database (https://phytozome-next.jgi.doe.gov/). Percent identity values for amino acid sequences were calculated with Clustal Omega and visualized with GraphPad Prism. Matrixes are colour coordinated based on the respective LDAP isoform and percent identity (zero to a hundred, white to designated isoform colour). VfLDAP1-3 are in bold.

Figure S2. Comparison of AlphaFold-predicted three-dimensional structures of the tung (V. fordii), Arabidopsis (A. thaliana), castor bean (Rinus communis), and cassava (Manihot esculenta) LDAP protein isoforms. The N- and C-termini for each protein are indicated, as well as their total predicted template modelling (pTM) values. The structures are locally coloured according to prediction confidence; with blue, white, and red coloring representing low, neutral, and high structural confidence, respectively (refer to key). Polypeptide sequences used for prediction models were obtained from Phytozome (refer to sequences in Table S1) and protein structures were visualized with PyMOL.

Figure S3. Heatmap depicting Pearson's correlation coefficients and statistical significance for the co-expression of select tung genes across seed development (i.e., 90 to 180 days Days-After-Pollination [DAP]) in relation to VfFADX. Data presented are based on the tung transcriptome database [3]; refer also to Figure 3B. Boxes are coloured according to Pearson's correlation coefficient (red, -1.0; white, 0; blue, 1.0; refer also to scale shown at the bottom) and statistically significant differences of at least $P \leq 0.05$ (as determined with two-sided student's t-tests) are indicated with an asterisk; crossed boxes identify correlation values of genes with themselves ($r = 1.0$). Statistical analyses were performed, and results were formatted into a heatmap using GraphPad Prism.

Figure S4. Confirmation of transient gene expression in N. benthamiana leaves using RT-PCR. mRNA was isolated from N. benthamiana leaves three days post-infiltration with the indicated constructs (i.e., VfLDAP1-3, VfFADX or VfDGAT2) or mock infiltrate (i.e., exclusively P19). Genomic DNA was isolated from non-infiltrated N. benthamiana leaves (Nb gDNA) as described in the 'Materials and Methods'. RT-PCRs were performed using gene-specific primers or with primers for N. benthamiana ACTIN, which served as an endogenous reference gene. Details on primers used for RT-PCRs are available in Table S2. RT-PCR products were analyzed by DNA gel electrophoresis and visualized with the RedSafeTM DNA

stain. Note the lack of RT-PCR products in all mock-infiltrated and Nb gDNA samples, confirming mRNA-specific amplification.

Figure S5. Effects of VfFADX, VfDGAT2 and/or VfLDAP ectopic expression on TAG content in N. benthamiana leaves. Total lipids were extracted from leaves transiently (co)expressing (as indicated with labels) either (A) VfFADX on its own or together with VfLDAP1-3, or (B) VfFADX alone or together with VfDGAT2 and/or VfLDAP1-3; mock samples were infiltrated with only the viral suppressor P19. Total lipids were then analyzed by TLC and GC-FID for total TAG content (μg/mg fresh weight [FW]). Values shown represent the means ± SD from three biological replicates (i.e., leaf materials harvested from three separate plant infiltrations). Letters in (A) and (B) represent statistically significant differences of at least $P \leq 0.05$ relative to the mock, as determined by Welsh's t tests.

Figure S6. Representative GC-FID chromatograms of methylated fatty acid (FAME) standards used in this study. Peaks correspond to the separation of (A) a FAME standard (i.e., 100 μg of Sigma-Aldrich CRM18918 F.A.M.E. mix), (B) tung seed oil FAMEs generated through the transmethylation of 100 μg of commercially-purchased tung oil (see 'Materials and Methods for additional details), and (C) a diluted combination of 100 μg CRM18918 F.A.M.E. mix and 50 μg of transmethylated tung oil. The temperature program, GC-FID method, and column details are described in the 'Materials and Methods'. Note in (B) and (C) the separation of eleostearic isomers (i.e., α-eleostearic acid [α-ESA] and β-ESA), as expected.
