# Supplementary figures and images for: Identification and Characterization of Lipid Droplet-Associated Protein (LDAP) Isoforms from Tung Tree (Vernicia fordii)
Source: Plants (Basel). 2025 Mar 5;14(5):814. doi: 10.3390/plants14050814 (PMC11901875; doi:10.3390/plants14050814)

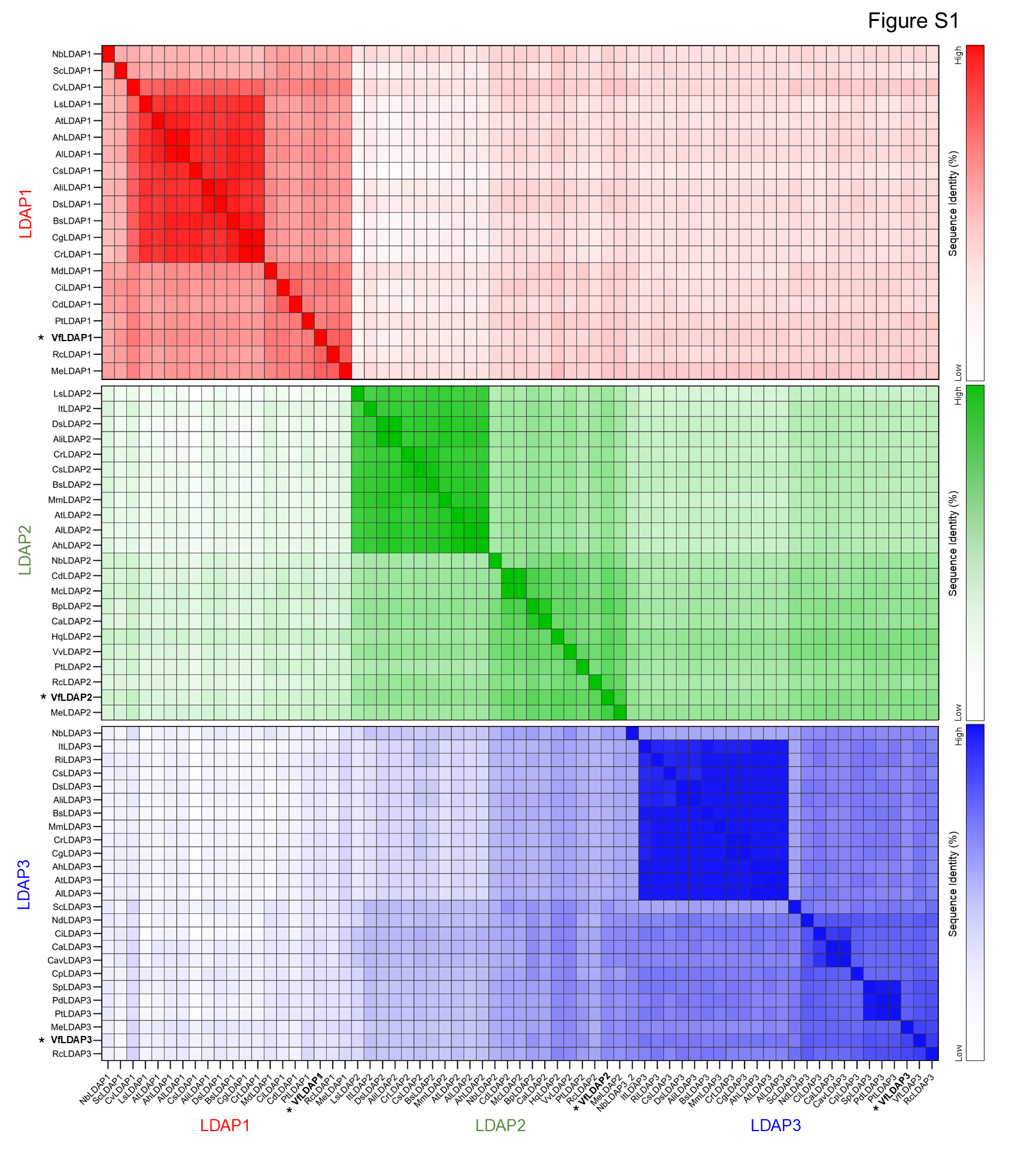

Supplement: Supplementary file 1 [file plants-14-00814-s001.zip › Figure S1.PNG]

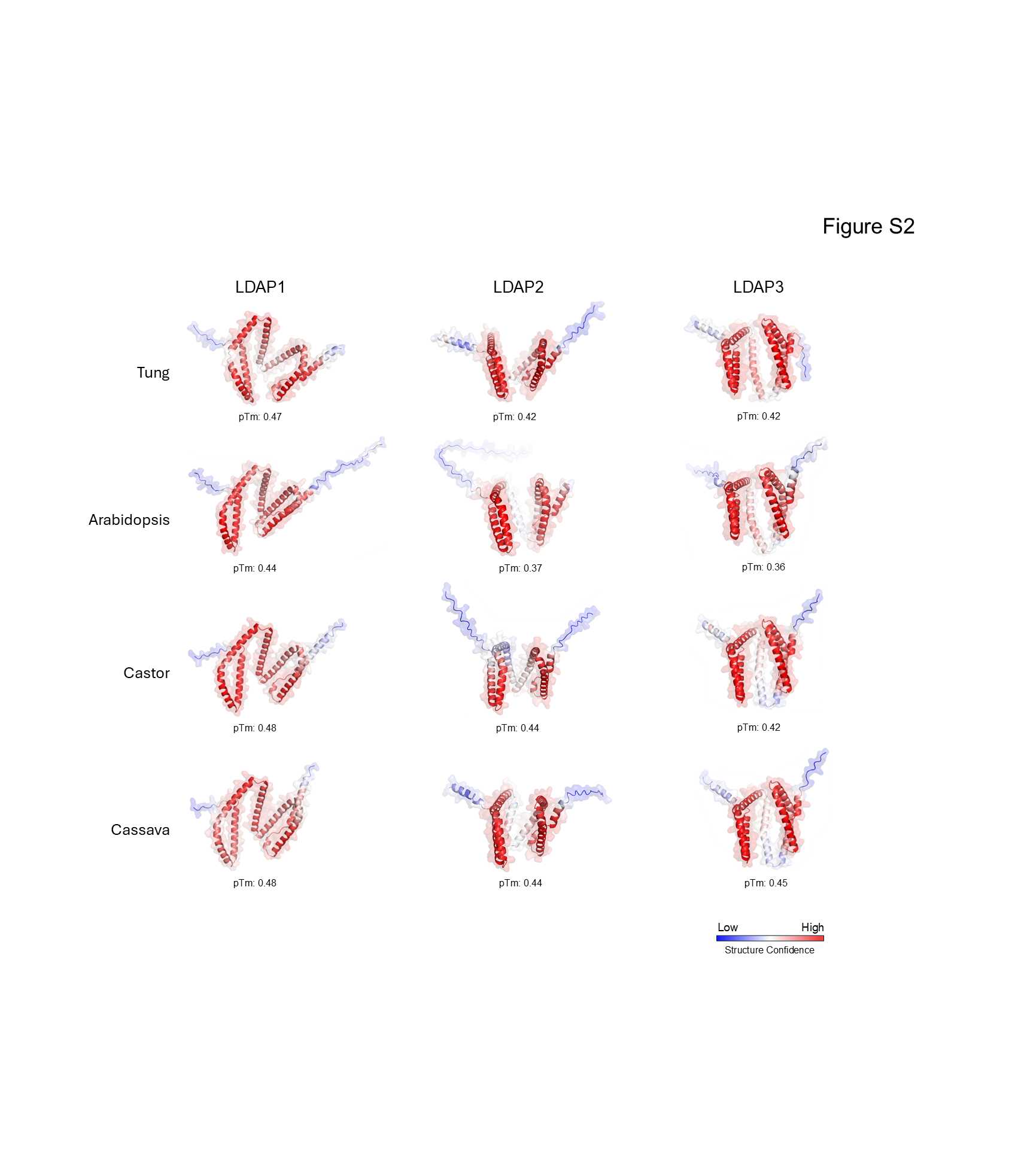

Supplement: Supplementary file 1 [file plants-14-00814-s001.zip › Figure S2.PNG]

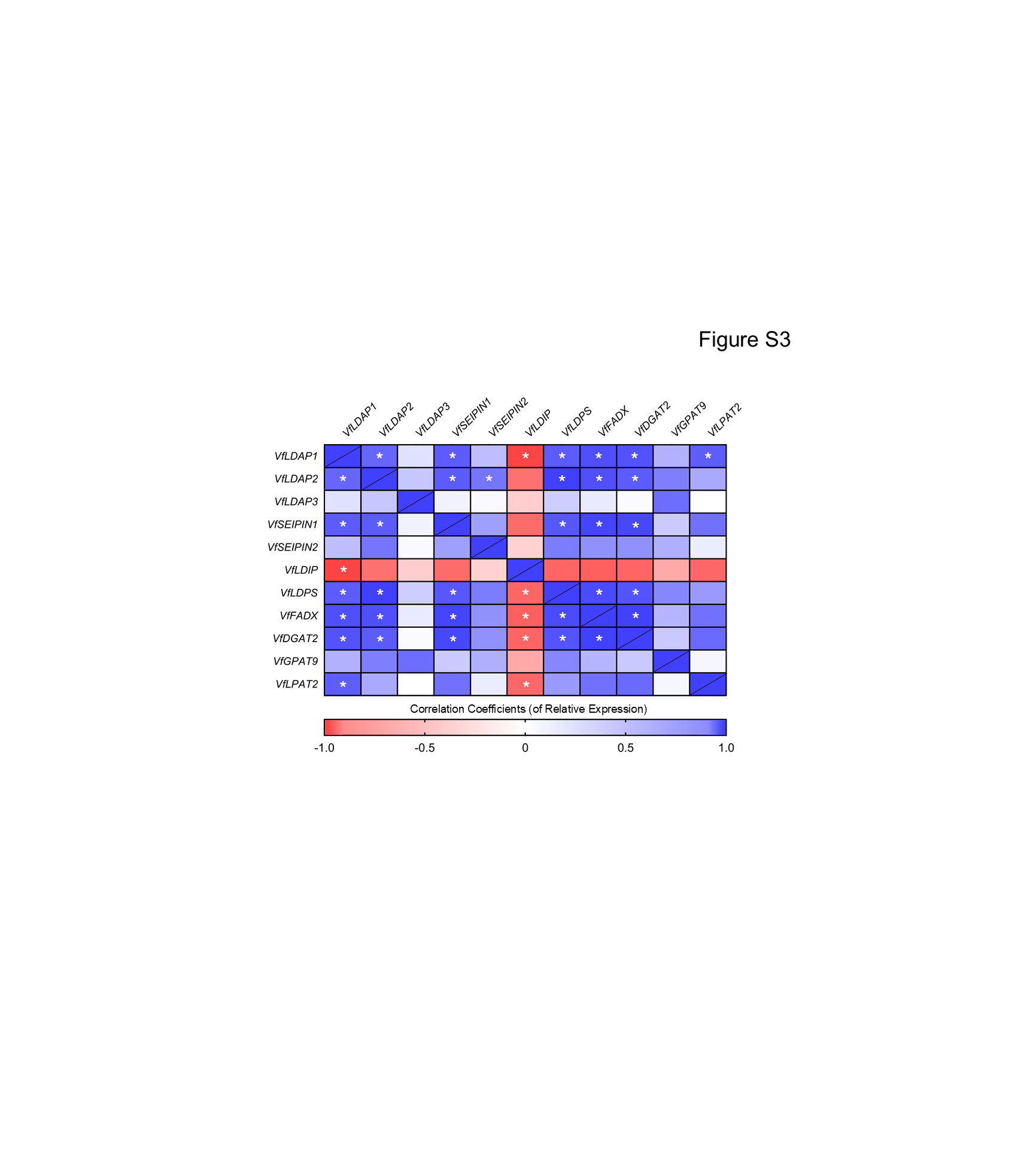

Supplement: Supplementary file 1 [file plants-14-00814-s001.zip › Figure S3.PNG]

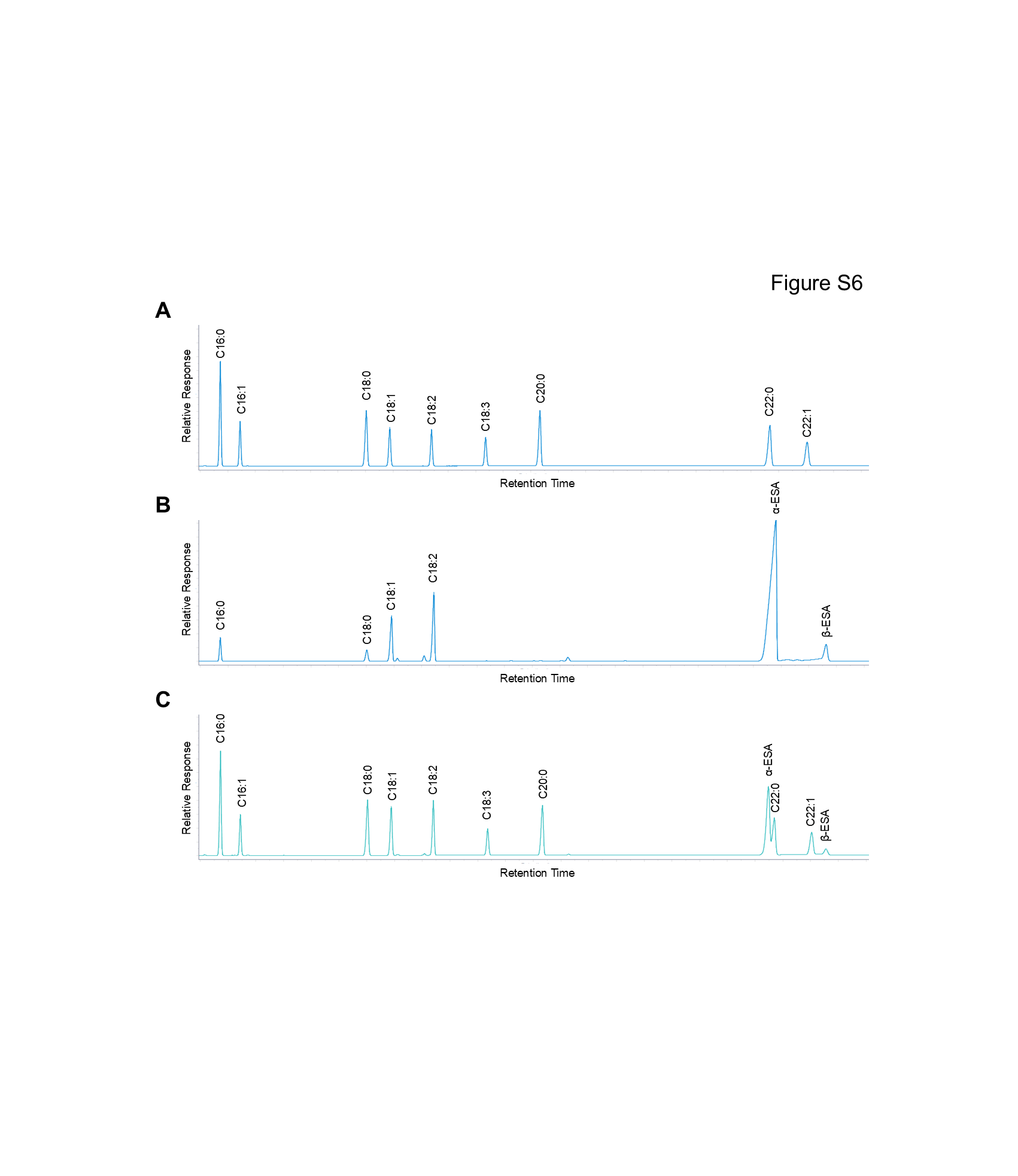

Supplement: Supplementary file 1 [file plants-14-00814-s001.zip › Figure S6.PNG]

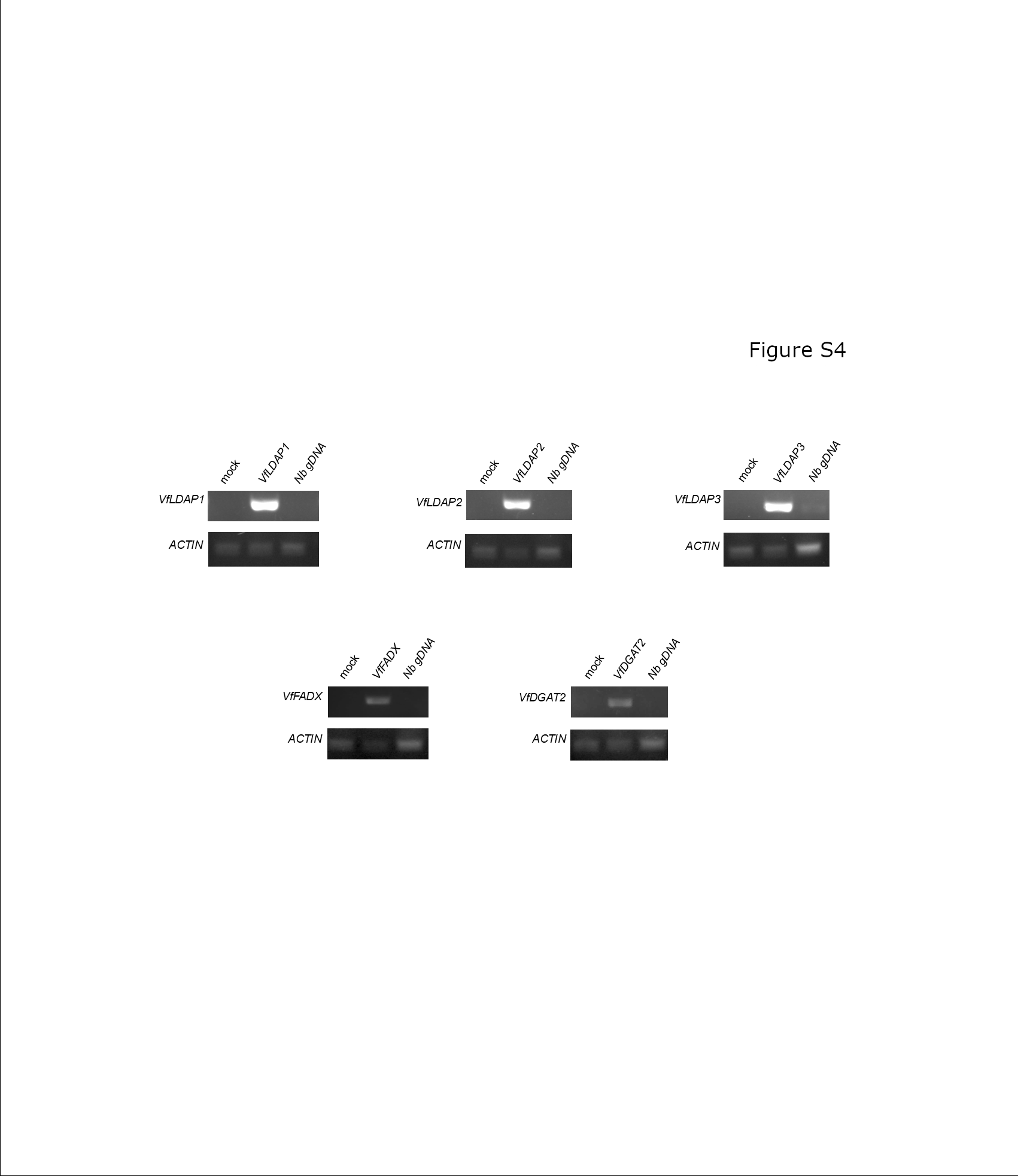

Supplement: Supplementary file 1 [file plants-14-00814-s001.zip › FigureS4.png]

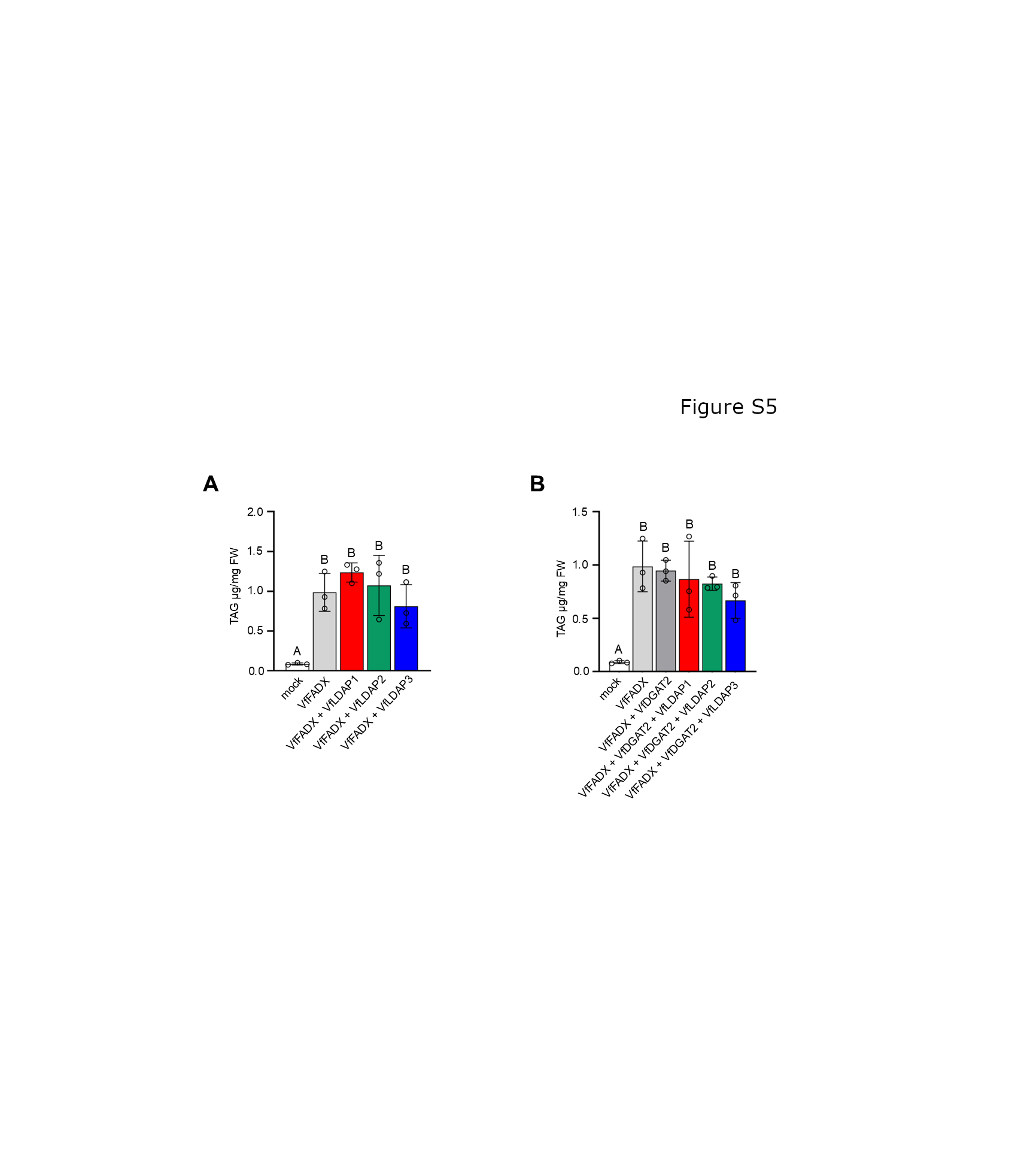

Supplement: Supplementary file 1 [file plants-14-00814-s001.zip › FigureS5.png]
